# Invasive Fungal Infections in the ICU: How to Approach, How to Treat

**DOI:** 10.3390/molecules19011085

**Published:** 2014-01-17

**Authors:** Elisabeth Paramythiotou, Frantzeska Frantzeskaki, Aikaterini Flevari, Apostolos Armaganidis, George Dimopoulos

**Affiliations:** Department of Critical Care Medicine, University Hospital ATTIKON, Medical School of Athens, Haidari 12462, Greece

**Keywords:** *Candida* infections, intensive care unit, fungi

## Abstract

Invasive fungal infections are a growing problem in critically ill patients and are associated with increased morbidity and mortality. Most of them are due to *Candida* species, especially *Candida albicans.* Invasive candidiasis includes candidaemia, disseminated candidiasis with deep organ involvement and chronic disseminated candidiasis. During the last decades rare pathogenic fungi, such as *Aspergillus* species, *Zygomycetes*, *Fusarium* speciesand *Scedosporium* have also emerged*.* Timely diagnosis and proper treatment are of paramount importance for a favorable outcome. Besides blood cultures, several laboratory tests have been developed in the hope of facilitating an earlier detection of infection. The antifungal armamentarium has also been expanded allowing a treatment choice tailored to individual patients’ needs. The physician can choose among the old class of polyenes, the older and newer azoles and the echinocandins. Factors related to patient’s clinical situation and present co-morbidities, local epidemiology data and purpose of treatment (prophylactic, pre-emptive, empiric or definitive) should be taken into account for the appropriate choice of antifungal agent.

## 1. Introduction

Great advances in contemporary medicine and especially in critical care achieved during the last decades have contributed not only to longer survival of patients, but also to the increasing incidence of opportunistic infections caused by fungi. Complex medical and surgical problems, disruption of natural barriers, multiple invasive procedures and prolonged antibiotic treatment are some of the factors contributing to the alarming increase of fungal infections in the Intensive Care Unit (ICU) setting [1,2]. The leading fungal infection is candidaemia. In 2007 the results of EPIC II study including 1,265 ICUs in 75 countries revealed that 19% of pathogens isolated in ICU patients were fungi [3]. *Candida* species (spp) were predominantly isolated (17%) followed by *Aspergillus* species. Candidaemia is associated with a high mortality and increased length of hospital stay and cost [4,5].^.^High attributable mortality may be due to delayed diagnosis and treatment, development of resistance or severity of illness. The purpose of the present review is to provide a practical approach to diagnosis and treatment of invasive fungal infections in the critically ill.

## 2. *Candida* Invasive Disease

*Candida* species are ubiquitous and constitute part of the normal human flora. Only a small percentage of the identified species cause disease in humans. *Candida* spp. is responsible for an extremely large spectrum of diseases [6,7]. Invasive *Candida* infections include candidaemia with or without endopthalmitis; disseminated haematogeneous infections; involvement of a single deep organ site (e.g. peritonitis, other abdominal infections, meningitis and infective endocarditis) and chronic hepatosplenic candidiasis mostly in haematological patients [8]. Source of *Candida* infection can be endogenous (gastro-intestinal flora or mucocutaneous colonisation) and exogenous (hands of healthworkers, contaminated infusates) even leading to local outbreaks [9]. Existing epidemiological data usually originate from candidaemia [10].

## 3. Incidence, Trends and Mortality

*Candida* bloodstream infections (BSIs) constitute the vast majority of nosocomial fungal infections. In a large nationwide surveillance study in United States (SCOPE) *Candida* spp were ranked fourth among the hospital-acquired BSIs (9%) [11]. The majority of infections (51%) were noticed in ICU [11]. Almirante et al showed an incidence of 4.3/100,000 population in Spain while in a study conducted in Iceland a rise from 4.3 to 5.7 cases per/100,000 population was noticed [12,13]. In another study including 106 institutions in seven European countries, the rates of candidaemia ranged from 0.20 to 0.38/1,000 admissions [14]. Limited data on the incidence of invasive *Candida* infections (ICI) in the critically ill are available. In an Australian study of ICU-acquired BSIs, *Candida* species were ranked fourth (15.5%) while according to Meyer *et al.* the incidence of primary nosocomial candidaemia in 682 German ICUs remained stable in a 5-year period [15,16]. In a recent study from Italy a total of 105 episodes occurred in an 18-month period with 16.5 cases per 1,000 admissions caused by yeasts and 2.3 cases per 1,000 admissions caused by filamentous fungi [17]. In a large prospective multicenter study conducted in French ICUs fungi were identified in 3.2% of patients with microbiologically documented infections [18]. Finally in an International Cohort Study (EUROBACT) of ICU-associated BSIs, candidaemia was ranked third below Gram-negative and Gram-positive isolates [19]. In a total of 1317 microorganisms, 98 (7,4%) were due to fungi and most of them due to *Candida* species [19]. The disparity between the results of studies may be explained by differences in demographic characteristics, variations in healthcare practice, difference in presence of co-morbidities and populations studied. Nevertheless the true incidence of invasive candidiasis may be higher not only due to the fact that the percentage of positive cultures lies between 30 and 50% but also because of the difficulty in diagnosing invasive candidiasis without candidaemia [20,21].

*Candida* BSIs are a well-recognized cause of morbidity and mortality among the critically ill. Though the crude mortality varies between studies, most authors report high percentages (39%–60%) and excess financial burden [11,22]. Attributable mortality is considered a better index to estimate the impact of ICI though it is more difficult to validate. A major difficulty is distinguishing between mortality attributed to candidaemia and mortality caused by the severe underlying disease. In a systematic review about candidaemia-associated mortality Falagas *et al.* included seven studies with attributable mortality ranging between 5 and 71%. For six of them a considerable difference in mortality between cases and controls was observed. Moreover the length of stay and hospitalization cost was significantly higher than that of controls [23]. If candidaemia is manifested with signs of sepsis or septic shock the associated mortality and financial burden are further increased [24]. 

## 4. The Changing Epidemiology of *Candida* spp.

Among *Candida* species which are pathogenic for humans, *Candida albicans* is the most frequently recognized followed by *C. parapsilosis*, *C. glabrata* and *C. tropicalis* [25]. Species infrequently encountered include *C. krusei*, *C*. *dublinensis*, *C. guillermondi*, *C. kefir*, *C. lusitaniae*, and *C. rugosa*. Nevertheless the list of species formerly considered as “non-pathogenic” is expanding with the increase in the number of vulnerable population and the ability of the laboratory to isolate new species.During the last decades a shift to non-*albicans* species has been noted [26]. According to data provided by an International Surveillance Program, ARTEMIS DISK, the distribution of *Candida* species in a 6-year period has changed with *C. albicans* decreasing by 10% though still remaining the most common isolated species [27]. This changing spectrum has been partly explained by the increasing prophylactic use of fluconazole though this hypothesis has not been confirmed by all investigators [28]. The increased proportion of non-*albicans* species has been also observed in critically ill patients with considerable differences in the percentage of *albicans* vs non-*albicans* spp [23]. In an Italian study 40% of the fungaemia episodes were due to *C. albicans*, followed by *C.parapsilosis* (23%), *C. glabrata* (15%), *C. tropicalis* (9%) and other species (13%) [29]. In a prospective national study conducted in 180 French ICUs *C. albicans* accounted for 57% of blood isolates followed by *C. glabrata* (16.7%), *C. parapsilosis* (7.5%), *C. krusei* (5.2%) and *C. tropicalis* (4.9%) [30]. Several risk factors have been associated with the presence of non-*albicans* species (NAS). In a case-comparator study Chow *et al.* showed that fluconazole exposure, presence of a central venous catheter and mean number of antibiotics per day were associated with an increased risk of BSIs due to NAS compared to *C. albicans* while in another study use of medical devices, steroids receipt and pre-existing candiduria were the risk factors associated with the presence of NAS [31,32]. Moreover every *Candida* species has its separate characteristics [7,10,33,34,35] (Table 1). The shift to non-*Candida* spp. is important because some of these species are resistant to fluconazole or other antifungal agents. Awareness of risk factors for the presence of NAS, rapid species identification and antifungal susceptibility testing are required for the proper and timely treatment of these infections.

**Table 1:** Main characteristics and factors affecting the emergence of *Candida* non-*albicans* spp [7,10,33,34,35].

*C. glabrata*	Most common in elderly patients
	Most common in malignancies
	Geographic variation
	Associated to the use of specific antibiotics, (piperacillin/tazobactam, vancomycin)
	Common in patients under TPN and with CVC
	Isolation system
	Solid organ transplantation
	Fluconazole exposure
*C. parapsilosis*	Nosocomial outbreaks
	Formation of biofilms in CVC
	Implanted devices
	TPN
	Less susceptible to echinocandins
	The second most common isolated strain in children
*C. tropicalis*	Haematological malignancies
	Neutropenia
*C. krusei*	Use of piperacillin/tazobactam, vancomycin
	Innate resistance to fluconazole
	Haematological malignancies
	Neutropenia
	Recent gastrointestinal surgery
	Fluconazole exposure
*C. guiillermondi*	Less susceptible to echinocandins
	Less susceptible to fluconazole
	Intravascular catheters

CVC = Central venous catheters, TPN = Total Parenteral Nutrition.

## 5. Laboratory Diagnostic Methods

In the critically ill patient the diagnosis of invasive candidiasis (IC) is not an easy task because signs and symptoms vary from minimal to dramatic while existing diagnostic procedures present several drawbacks. Blood cultures (BCs) remain the cornerstone of diagnosis but they have suboptimal sensitivity (30%–50%) and need long incubation time [21]. Moreover in deep-seated candidiasis or in patients under fluconazole prophylaxis, BCs are often negative [36,37]. Newer culture methods have raised the sensitivity of *Candida* detection to almost 70%, but they require a minimum of 24 to 48 h to become positive and thus their result may come late in the course of the infection [38].

In order to make timely diagnosis more feasible, several serological markers have been developed. These tests consist of the detection of various agents, components of the fungal cell wall such as the mannan and 1,3-β-d-glucan tests whereas antibodies against the mannan antigen (anti-mannan) have also been developed [21,39,40,41]. For the detection of mannan antigen two methods have been described: a latex agglutination test (Pastorex-*Candida*; Biorad, Biorad laboratories GmBH); and an enzyme–linked immunosorbent assay (ELISA)(Platelia–*Candida*; Biorad). Both methods have equal specificity (90%–100%) in diagnosing candidaemia [38,42] but ELISA has superior sensitivity, ranging between 30%–60%, depending on study population, diagnostic cut–off points and *Candida* species under consideration, being higher for *C. albicans* [42,43,44]. In high risk patients, performing the test two to three times per week is highly recommended, since its circulation in the bloodstream is intermittent [21,38]. Combined detection of mannan and anti–mannan antibodies in blood (ELISA) offers higher sensitivity (80%–100%) but concurrent drop in specificity (84%) [39,42]. The detection of 1,3-β-d-glucan (BG) (Fungitell Assay; Cape Cod, MA, USA), which is a pan-fungal marker (except for *Mucorales* and *Cryptococcus*), demonstrates variable sensitivity depending on the cut-off diagnostic value and the *Candida* species under consideration [40,41,44]. One single positive test is only suggestive of *Candida* infection and it must be interpreted with caution due to false positive results from albumin and/or immunoglobulin administration, haemodialysis or Gram-positive bacteraemia [38,45]. A negative BG test is associated with high negative predictive value (>90%) and can be used to rule out IC. In a recent study by Posteraro *et al.* the BG test exhibited excellent positive (72,2%) and negative (98,7%) predictive values in critically ill patients [46]. An indirect immunofluorescence assay has also been developed for the detection of antibodies (IgG) against *C. albicans* (*C. albicans* germ-tube antibodies, CAGTA, Vircell, Granada, Spain) showing 77%–89% sensitivity and 91%–100% specificity [45,47]. Though excellent for monitoring therapy, it cannot discriminate BSIs among *Candida* species and it has not been validated in the critical care setting.

Additionally nucleic acid-based detection methods (real-time polymerase chain reaction) have been developed for the detection of *Candida* infection [36,48,49]. According to Ngyuen *et al.*, both real–time PCR and BCs have comparable sensitivities in diagnosing candidaemia (60%) but PCR is invaluable in diagnosing deep–seated candidiasis with negative BCs (sensitivity 88% for PCR vs 17% for BCs) [36]. Moreover PCR performance is not influenced by the addition of antifungal therapy [36]. Data concerning the use of PCR in the critical care setting are limited. McMullan *et al.* tested three real–time PCR assays for the detection of *Candida* spp in non-neutropenic critically ill patients with candidaemia, showing excellent sensitivity (90.9%) and specificity (100%) [48].

Finally several recently introduced diagnostic tools include: (a) matrix- assisted laser desorption ionization—time of flight mass spectrometry (MALDI–TOF MS). The test seems a rapid and reliable tool for the identification of yeasts and yeast-like fungi [50]; (b) a commercial assay allowing the molecular detection of fungi in blood (Lightcycler Septofast—Test, Roche Diagnostics GmBH, Mannheim, Germany) and (c) a commercial assay based on fluorescence *in situ* hybridization (PNA FISH). This method is a rapid way to differentiate among the usually isolated *Candida* species in blood provided that blood cultures have developed *Candida* spp [51]. In a recent study, comparison between MALDI–TOF and PNA–FISH for yeasts showed that the PNA FISH assay had a 100% agreement with the result obtained by MALDI-TOF MS [52]. 

## 6. Risk Factor Assessment

Invasive *Candida* infection leads to increased mortality in case of treatment delay. The lack of methods with a high sensitivity and specificity for the timely diagnosis of IC created the need for the identification of risk factors and evaluation methods in order to ensure timely treatment even in the absence of laboratory evidence of infection. These risk factors have been described in many studies [53,54,55,56] and they are summarized in Table 2.

**Table 2:** Factors leading to *Candida albicans* invasive infections in ICU patients [53,54,55,56].

Prolonged ICU stay
Treatment with corticosteroids
Diabetes mellitus
Advanced age
Central venous catheter
Gastrointestinal surgery
Total parenteral nutrition
Prolonged antimicrobial use
Pancreatitis
Immunosuppressive agents
Chemotherapy
High disease severity score (APACHE II > 20)
Neutropenia
Renal replacement therapy
Malnutrition
Multiple site colonisation
Burns over 50% of body sites
Major trauma

Fungal colonisation has been associated with the development of IC, yet according to recent data, a small proportion (3%–25%) of colonised patients subsequently develop invasive disease [57,58]. Due to the time-consuming nature of all the aforementioned laboratory methods and since in IC immediate antifungal drug therapy is mandatory, several researchers have investigated *Candida* colonisation patterns in combination with the established risk factors of Table 2, in order to identify specific patient groups who might benefit from antifungal prophylaxis/therapy. These “risk factors” expressed as *Candida Prediction Rules* have been established in many studies [56,58,59,60,61,62,63,64] and they are summarized in Table 3.

**Table 3:** *Candida* Prediction Rules.

*Authors*	*Aims and Criteria*	*Factors and Prediction Rule*
Leon [56]	Surgical patients	“*Candida* score” Factors leading to invasive candidiasis development include *multifocal colonisation, surgery on ICU admission, severe sepsis, TPN.* A “Candida score” > 2.5 selects the non-neutropenic ICU patients who might benefit from early antifungal treatment
Agvald-Ȍhman [58]	To identify patients at risk of IC among those with a length of ICU stay of at least 7 days	*Candida* colonisation index ≥ 0.8 and *recent extensive gastroabdominal surgery*
Pittet [59]	Surgical and neonatal ICUs To identify patients in surgical and neonatal ICUs at increased risk of subsequent infection.	*CCI = Candida* Colonisation Index. Patients with CCI ≥ 0.5 at high risk.
Dupont [60]	Patients with severe peritonitis	Presence of at least three of *female sex, cardiovascular failure, upper gastrointestinal tract origin, ongoing antimicrobial therapy* predicts yeast isolation in the peritoneal fluid
Ostrosky-Zeichner [61]	To identify patients at increased risk of IC in medical and surgical ICUs	Any systemic antibiotic (days 1–3) ORCVC (days 1–3) **AND at least TWO of the following:** *TPN (days 1–3), any dialysis (days 1–3), any major surgery (days 7–0), pancreatitis (days 7–0), steroid use (days -7–3). other immunosuppressive drug (days 7–0)*
Hermsen [62]	Due to the high Negative Predictive Value, the rule applies best to identify patients who are LEAST likely to benefit from antifungal therapy	*Current systemic broad-spectrum antibiotic use, CVC, TPN, abdominal surgery within last 7 days, steroid use, hospital LOS*
Paphitou [63]	To identify patients at increased risk of invasive candidiasis in surgical ICUs	Presence of *new onset hemodialysis, TPN, diabetes mellitus and broad-spectrum antibiotics* predict invasive candidiasis
Ostrosky-Zeichner [64]	To identify patients at increased risk of invasive candidiasis in the ICU	Mechanical ventilation (days 1–3) **AND** CVC (days 1–3) **AND** at least **ONE** of the following *TPN (days 1–3), any dialysis (days 1–3), any major surgery (days 7–0), pancreatitis (by CT or lipase >1000iu, days 7–0), steroid use (>1dose of prednisone equivalent to 20mg, days 7–0), other immunosuppressive drug (>1 dose, days 7–0)*

CVC = Central venous catheter, LOS = Length of Stay, TPN = Total parenteral nutrition.

## 7. Aspergillus

*Aspergillus* spp are moulds which are able to cause life-threatening invasive disease in immunocompromised individuals and local disease in immunocompetent persons. The latter can present with a spectra ranging from localised infection of the lungs and sinuses to allergic reactions due to spore inhalation. The species present a worldwide distribution and are found in the environment, plants and decomposing organic matter. Among the hundreds of *Aspergillus* species few are able to cause disease to humans. The most commonly encountered include *A. fumigatus* followed by *A. flavus* and *A. terreus*. The epidemiology of aspergillosis in the ICU is difficult to establish due to the inhomogeneity of hospitalised patients, the diagnostic difficulties necessitating a biopsy and the difficulty in discriminating between colonisation and disease [65]. Sometimes autopsy is necessary to prove the diagnosis [66] while a high mortality is reported [67]. Possible sources of *Aspergillus* in the ICU include improperly cleaned ventilation systems, water systems, or even computer consoles [68]. ICU patients with impaired immunity are prone to develop the invasive form of the disease in lungs and sinuses. Neutropenic patients usually develop the aggressive angioinvasive form while patients under steroid treatment present with a cavitating lesion [65]. Anastomotic regions are the fungus target in patients with lung transplantation [69] while rarer presentations such as endocarditis or osteomyelitis have been described [70,71].

The diagnostic approach includes evaluation of possible risk factors, clinical and radiological signs and the implementation of specific laboratory and “high technology” methods.

Persons at greatest risk include patients with haematological malignancies, solid organ transplant recipients, haematopoietic stem cell transplant recipients (HSCT) but also prolonged steroid treatment before ICU admission, chronic obstructive pulmonary disease, severe burns, previous cardiac surgery *etc.* [65]. Clinical signs and symptoms are not specific e.g. fever not responsive to antibiotics and signs of nosocomial pneumonia. Haematogenous dissemination of *Aspergillus* to the brain can cause seizures, brain infarctions, intracranial haemorrhage and meningitis.

Radiologic chest radiograph might be negative at the beginning of the disease, or might show nonspecific changes even during the late stages [72] and they include single or multiple small nodules with the “halo sign”, a zone of low attenuation with a translucent ground-glass halo around, more frequently appearing in neutropenic patients [73]. Vandewoude *et al.* recorded nonspecific infiltrates and consolidation as the commonest radiological findings in critically ill patients with Invasive Aspergillosis (IA) [74].

The gold standard of diagnosis of IA is the histopathological identification of *Aspergillus* in invasive tissue sampling [75]. However, biopsies are rarely performed since critical illness is often associated with coagulation abnormalities, while neutropenia is often accompanied by thrombocytopenia. Moreover the isolation of *Aspergillus* from cultures of bronchial secretions, broncho alveolar lavage (BAL) or other body fluids or tissues, might represent colonisation rather than infection [76]. However, their positive predictive value is higher in immunosuppressed patients [77]. Focusing on the critically ill the identification of *Aspergillus* in respiratory samples lies between 10%–80%, according to different studies [78,79].

Two non-culture-based diagnostic techniques are available: the detection of galactomannan (GM) and β-d-glucan, two fungal cell wall components in blood or other body fluids. The first is released in body fluids during *Aspergillus* growth [72]. It can be detected in serum and BAL, notably before the clinical manifestation of IA [80]. This method leads to the calculation of the galactomannan index (GMI), which is considered to be evidence of IA if it exceeds a certain threshold. However there are false positive results attributed to the concomitant use of β-lactam antibiotics, to the presence of dietary source of galactomannan (cereals, pasta) or to cross reactivity with other antigens [81]. In non-neutropenic patients the lower sensitivity of the method might be the result of rapid clearing of GM by neutrophils from the circulation [81]. Variable results have been reported in the measurement of GM in BAL and urine and excellent results from cerebrospinal fluid. The reported sensitivity and specificity of GM results in BAL reaches 88% and 86% respectively [82]. As for β-d-glucan, false positive results might be associated to the use of immunoglobulin contaminated with fungal products, the presence of bacterial infections, the administration of several antibiotics and the use of cellulose filters of haemodialysis [83].

An additional method for IA diagnosis is PCR [84]. The sensitivity and specificity of PCR in BAL fluid is 67%–100% and 55%–95%, whereas in serum the values are 100% and 65%–92% respectively [85,86]. A possible drawback of PCR diagnostics is the difficulty in discrimination between colonisation and infection [87]. However, according to Li *et al.*, a whole blood quantitative real time PCR (qPCR) targeting to a special gene sequence of the fungus could prove as a clinically reliable technique for diagnosis of IA [88]. Additionally, it has been suggested that qPCR is a comparable to GM diagnostic method for IA, and that combining qPCR with GM is a more scientific and sensitive approach to IA diagnostics [89].

The European Organization for Research and Treatment of Cancer/Mycosis Study Group (EORTC/MSG) has incorporated the risk factors, clinical signs and laboratory tests in an attempt to stratify the diagnosis of invasive pulmonary aspergillosis (IPA) into *proven*, *probable*, and *possible* [90]. Histopathologic or cytopathologic examination of a proper specimen is a prerequisite for the *proven* diagnosis. *Probable* IPA is confirmed by a combination of clinical and host factors and positive mycological criteria, including cultures or detection of cell wall components. *Possible* IPA diagnosis is based on the presence of clinical and host features but without positive mycology. However, the aforementioned criteria have been validated in immunosuppressed patients, while in critically ill without classic risk factors this classification has been questioned [74]. Therefore, Blot and colleagues proposed a clinical diagnostic algorithm, deriving from EORTC/MSG in order to diagnose IPA and discriminate colonisation from infection [91]. According to that algorithm, IPA is considered to be *probable (“putative”)* if there are compatible signs, abnormal medical images and either host risk factors or BAL culture positive for *Aspergillus*. This simple clinical algorithm has been validated in critically ill patients with histopathologically proven aspergillosis, showing specificity 61% and sensitivity 92%, and might prove to be useful in the IA diagnostics in critically ill patients.

## 8. Mucorales

The class of Zygomycetes is divided in two orders, *Entomophtorales* and *Mucorales*. *Entomophtorales* are rarely implicated in a life-threatening angioinvasive human disease, whereas *Mucorales* are responsible for mucormycosis, the third commonest invasive fungal infection [92]. *Mucorales* are traditionally divided in six families: *Mucoraceae*, *Cunninghamellaceae*, *Saksenacea*, *Thamnidaceae*, *Syncephalastraceae* and *Mortierellaceae*. Recently a seventh family, called *Absidiaceae*, was added. *Rhizopus*, *Mucor*, *Rhizomucor*, *Absidia*, *Apophysomyces*, members of the genera of the two families of *Mucoraceae*-*Absidiaceae*, are pathogens most commonly implicated in human disease [93].

These organisms are ubiquitous saprophytes in nature rarely infecting organisms with intact immune system. Based on anatomic localization, mucormycosis can be classified in six forms: rhino- cerebral, pulmonary, cutaneous, gastrointestinal, disseminated and uncommon presentations [94]. Clinical suspicion arises from the patient’s medical history and physical examination. All patients with a chronic infection of the paranasal sinuses, burn or trauma wound infection should have their skin meticulously examined for the presence of either black discoloration or black eschars. A hallmark of the disease is propagating tissue necrosis due to vascular invasion by the fungus, though absence of black discoloration should not exclude the diagnosis. The microbiological laboratory must be notified in order to perform direct mycological examination and culture of a histopathological specimen.

Sporadic mucormycosis is a life - threatening condition, always associated with certain risk factors, mainly neutropenia and prolonged acidosis of either diabetic or renal origin. Mucormycosis in the ICU setting is related more commonly to massive injuries e.g. from motor vehicle accidents, natural disasters [95,96] or complex combat trauma [97]. There has also been one report from a Spanish ICU of an outbreak of gastrointestinal mucormycosis due to wooden tongue depressors that had been contaminated by two *Rhizopus* species [98]. 

The diagnosis is based on the examination of tissue samples. Stains of fixed tissues with hematoxylin-eosin, Grocott-methanamine-silver (GMS) or periodic acid-Schiff (PAS) are pathognomonic, showing broad nonseptated hyphae, irregularly branched at angles varying from 45–90° [99].Vascular invasion of the lesion and necrosis accompany the infective process. Blood cultures can be obtained but they are seldom positive. Immunochemical tests such as 1,3-β-glucan or galactomannan are not useful. PCR for the detection of DNA of certain *Mucorales* species has been recently described, but it cannot be routinely used [100,101]. Table 4 summarizes the techniques available for the diagnosis of *Aspergillus* and *Mucorales* spp*.*

**Table 4:** Diagnostic approach for invasive Aspergillosis and *Mucorales* infections in the ICU.

Diagnostic method	*Aspergillus*	*Mucorales*
Histopathology	Definite diagnosis	Definite diagnosis
Radiology	No specific findings	No specific findings
Blood cultures	Extremely rare	Extremely rare
Respiratory samples cultures	Sens: 10%–80%	Sens: 67%, Spec: 100% (BAL)
qPCR blood-BAL	Sens: 67%–100%, Spec: 55%–95%	Sens: 40%–90%, Spec: 100%
Antigen assays	Galactomannans (GMI) ^1 ^> 0.5 Sens: 71% (BAL 88%), Spec: 89% ^1^ (BAL 86%)	Investigational
	1,3-β-glucan “Panfungal marker”	No
Algorithms	EORTC/MSG criteria ^2^ Vandewoude and collegues ^3^ Sens: 61%, Spec: 92%	

Sens = Sensitivity, Spec = Specificity, ^1^ in patients with haematological cancer or haematopoietic stem cell transplant recipients; ^2^ validated in immunosuppressed patients, ^3^ in critically ill patients

## 9. Antifungal Compounds

Recent decades have seen an impressive progress in the development of our therapeutic antifungal armamentarium. Four classes of antifungal drugs are currently available for the treatment of invasive fungal diseases in critically ill patients. They include: (1) polyenes, (2) azoles, (3) echinocandins and (4) pyrimidine analogues.

### 9.1. The Role of Polyenes/Amphotericin B

For several decades, amphotericin B deoxycholate (AmB) has been the mainstay of treatment for invasive fungal infections. It possesses a broad spectrum of activity against not only most of *Candida* species- with the exception of *C lusitaniae* and *C guillermondii* but also *Cryptococcus neoformans* and the *Mucorales*.The antifungal spectrum of the drug also includes filamentous fungi especially *Aspergillus* spp (with the exception of *A. terreus*) dimorphic fungi such as *Histoplasma*, *Blastomyces*, *Coccidioidomyces*, *Paracoccidiodomyces*, and emerging yeasts such as *Trichosporon* spp and *Geotrichum* spp although *Trichosporon* strains with high MICs to amphotericin B were detected in some studies [102]. Its efficacy lies upon its fungicidal properties. The drug interacts with the membrane sterol, increases the permeability of the cell membrane and allows leakage of cell components ending to the death of fungus cell [103,104]. Despite many years of clinical use, resistance development is unusual except for *C. glabrata* and *C. krusei* isolates presenting higher minimum inhibitory concentrations (MICs) [105]. On the contrary filamentous fungi exhibit increasing resistance to polyenes [106]. *A*. *fumigatus*, the species exhibiting usually susceptibility to amphotericin B, has shown an increase in resistance [107]. The use of conventional AmB is limited due to a narrow therapeutic window and significant adverse effects, especially nephrotoxicity [108] The risk of nephrotoxicity increases significantly with the concomitant use of other nephrotoxic medications. Three lipid formulations of amphotericin B have been developed, namely liposomal amphotericin B (LipAmB), amphotericin B lipid complex and amphotericin B colloidal dispersion, which retain the activity of the parent drug [109,110] and exhibit a better safety profile [111] but have an increased cost compared to AmB. Nevertheless, side effects such as anaemia, thrombocytopenia, nephrotoxicity and hepatotoxicity have been reported with LipAmB, the first two of them presenting in a dose-dependent manner [112]. Moreover it seems that LipAmB is less toxic than the other two [113]. Infusion-related reactions including fever, rigors, chills, myalgia and headaches are also a major problem of AmB use. Slow infusion rates or pretreatment with acetaminophen or hydrocortisone can blunt these reactions which are less frequent or severe when newer lipid formulations are used [109]. In contrast with the 2004 guidelines, the 2009 IDSA guidelines consider AmB and its lipid formulations as *alternative* agents for the treatment of candidaemia [114] due to the associated nephrotoxicity and not to a diminished therapeutic result [115]. This recommendation has been subject of criticism; in a recent paper, Povoa and Pereira expressed their concern about published data upon which recent guidelines have been based [116]. Dreyfuss and collaborators also claim that there is enough body of evidence to support that if prevention measures are taken (adequate hydration, electrolyte repletion and continuous infusion instead of rapid administration) nephrotoxicity is not a problem while the efficacy remains the same with new treatments [117].

### 9.2. Azoles

Four azole compounds are available for the treatment of invasive fungal diseases: itraconazole, fluconazole, voriconazole and posaconazole.

#### 9.2.1. Itraconazole

The older agent of azoles, with a good activity against *Aspergillus* spp. Once available as oral agent, it is now available in parenteral form as well. The role of itraconazole in critically ill patients with invasive fungal infections (IFI) has not been determined.

#### 9.2.2. Fluconazole

Fluconazole is active against most species of *Candida* with the exception of *C. glabrata* and *C. krusei* but not against *Aspergillus* or *Zygomycetes*. Fluconazole acts by inhibiting an enzyme necessary for the biosynthesis of cell membrane sterol ergosterol [118] and although it is fungistatic [119,120] it has a proven efficacy for invasive candidiasis in the ICU. The drug is available in both IV and oral formulation, possesses a good safety profile and a low cost. Consequently it is the one of most commonly prescribed antifungal drugs in the ICU. Nevertheless it presents some clinically significant drug interactions such as increased concentrations of cyclosporin, tacrolimus, warfarin, carbamazepin and rifampicine [121]. Especially in the ICU setting important drug - drug interactions concern the usually administered agents fentanyl (anopiate) and midazolam (a sedative benzodiazepine). Because these drugs are extensively metabolized by the isoenzyme CYP3A4/5, co-administration of fluconazole with fentanyl or/and midazolam increases serum levels of those two drugs by competitive inhibition [122] Skrobik *et al* have shown that levels of fentanyl and midazolam are increased with the co-administration of fluconazole or voriconazole [123]. Elevation of hepatic transaminases and symptoms from the gastrointestinal tract are the commonest adverse effects.

#### 9.2.3. Voriconazole

Voriconazole is a second-generation member of azole family. It possesses a broad spectrum of activity covering *Candida* species including the ones resistant to fluconazole, *Cryptococous neoformans*, *Aspergillus* and *Fusarium* species. *Zygomycetes* are not susceptible to voriconazole. It is the drug of choice for invasive forms of aspergillosis [124]. It acts by inhibiting the ergosterol synthesis of cell membrane by fungi. It is fungistatic for yeasts but fungicidal for some filamentous fungi [125,126,127,128]. Immunomodulatory properties against *A. fumigatus* have also been ascribed to voriconazole [121]. Adverse effects include reversible visual disturbances, confusion, hallucinations, rash and hepatitis. It can be given both orally and intravenously [129,130] but the intravenous form should not be used in patients with creatinine clearance of <50 mL/min or under haemodialysis due to the possible accumulation of cyclodextrin (a solvent vehicle with potential toxic effects) [131] though the studies’ results are not uniform [132]. A wide variability in plasma concentrations of the drug make therapeutic drug measurements an important parameter for achieving a therapeutic goal while minimizing adverse effects [133,134]. Factors affecting drug levels include age, interactions with other drugs (tacrolimus, sirolimus), liver disease, and genetic polymorphism of the cytochrome CYPZC19. In the ICU setting drugs commonly given and interacting with voriconazole include omeprazole, phenytoin and warfarin. Another important drug interaction includes the co-administration with fentanyl and midazolam, two commonly used drugs in the critically ill patients, the levels of which increase if voriconazole is administered [122,123]. Voriconazole has been successfully used in the ICU setting as salvage treatment in patients with IFIs previously treated with azoles [135].

#### 9.2.4. Posaconazole

The newest compound of azoles is active against *Candida* spp, *Aspergillus* spp, *Cryptococcu*s and *Mucorales*. It is available only in oral suspension form (better absorbed when given with a fatty meal) therefore its use in the critically ill is very limited. Posaconazole is given to prevent the fungal infections in neutropenic patients with leukaemia [136]. The compound is also approved as second-line agent for invasive aspergillosis and considered as partially effective for mucormycosis [137].

### 9.3. Echinocandins

This is the newest class of antifungal agents developed and they include caspofungin, micafungin and anidulafungin. They possess a limited spectrum of activity covering only *Candida* and *Aspergillus* species. Their mode of action is the inhibition of the synthesis of 1,3-β-glucan, a polysaccharide which maintains the integrity of the cell wall and they are fungicidal *in vitro* against *Candida* but fungistatic for *Aspergillus* [138]*.*

They are given only intravenously in a slow infusion rate in order to avoid the rare infusion - related reactions. Since the active drug is not excreted into the urine their use in candiduria is not suggested. Moreover, the level of echinocandins in special compartments such as cerebrospinal fluid and intraocular compartment remains low. *C. parapsilosis* is associated with higher MICs and therefore echinocandins are not recommended for candidaemia caused by these species. Their major advantages are the few or negligible interactions with other drugs, especially for micafungin and anidulafungin and their minor adverse effects (abnormal liver function tests, phlebitis, or histamine-like reactions) [139]. Differences between echinocandins are minor [140,141,142] (see Table 5).

A significant advantage of echinocandins is that being fungicidal they offer much more rapid resolution of symptoms with fewer complications when compared to fluconazole [143]. Due to their favorable therapeutic and safety profile, echinocandin use in the critically ill exhibited a rapid increase and this drug class was established in guidelines as primary treatment option for invasive candidiasis. Increasing use of echinocandins during the last years has raised fears about emergence of resistance but for the present time this phenomenon remains rare [141]. Another concern is the high cost of treatment. Table 6 summarizes the spectrum of antifungal drugs, suggested dose in IC and need of dose adjustment in renal failure.

### 9.4. Pyrimidine Analogues

Flucytosine is the main representative of the class. It is available only as oral formulation in USA but also in intravenous form in other countries and it is mostly used in combination with AmB for special forms of invasive candidiasis *(Candida* endocarditis, meningitis, or urinary tract candidiasis) or other severe mycosis such as cryptococcosis, aspergillosis and chromoblastomycosis [144]. 

## 10. Future Therapeutic Options

Newer drugs under investigation include isavuconazole, ravuconazole and albaconazole while a human recombinant monoclonal antibody (Mycograb–Neutec Pharma, Manchester, UK) has been used in combination with AmB showing favorable results [8,145].

**Table 5:** Differences on side effects and drug-drug interactions of echinocandins.

Caspofungin	Micafungin	Anidulafungin
Some interactions with rifampin, phenytoin, carbamazepine, antiretroviral agents and dexamethasone [ 140]	Lacks efficacy and safety data in patients with severe hepatic impairment.	Does not undergo any degree of hepatic or renal metabolism
Interactions with cyclosporin A (liver function abnormalities) [ 141]	Reported formation of liver tumors in rodents rose some concern about its use ( in humans no similar effects have been shown) [ 142]	No dose adjustement is necessary in patients with hepatic or renal impairment

**Table 6:** Spectrum of antifungal drugs, usual dose in invasive infections and dose adjustement in renal failure.

Antifungal drug	*C. albicans*	C. *parapsilosis*	*C. glabrata*	*Aspergillus*	*Mucorales*	*Cryptococcus*	Dose	Dose in Renal failure
AmB	S	S	S	S *	S	S	0.5–1 mg/kg/d	same
lipAmB	S	S	S	S *	S	S	3–5 mg/kg/d	same
Fluconazole	S	S	SDd - R	R	R	S	800 mg (12 mg/kg) l d 400 mg (6 mg/kg)	Adjustment of the dose
Itraconazole	S	S	SDd - R	S	R	Ms	200 mgIV/bid 2 d then 200 mg/d	same
Posaconazole	S	S	SDd - R	S	S	S	200 mg qid	Same in mild, moderate
Voriconazole	S	S	SDd - R	S	R	S	400 mg/bid then 200 mg/bid	IV not given inCrcl < 50 mL/min
Flucytosine	S	S	S	R	R	S	50–150 mg/kg in 4 doses	Adjustment of the dose
Caspofungin	S	Rc	S	S	R	R	70 mg/d l dthen 50 mg/d	same
Micafungin	S	Rc	S	S	R	R	100 mg/d	same
Anidulafungin	S	Rc	S	S	R	R	200 mg/d l d, then 100 mg/d	same

* Not all *Aspergillus spp* susceptible to amphotericin B deoxycholate or liposomal amphotericin B. Abbreviations: S: susceptible, R: resistant, Rc: resistance depending on the concentration due to higher MICs of *C.parapsilosis* to echinocandins, SDd – R: susceptible dependent on dose, AmB: amphotericin B deoxycholate, lip Am B: liposomal amphotericin B, ld: loading dose, bid: twice daily, qid: four times daily, S:susceptible, R: resistant, Ms: modest activity, IV: intravenous form, Crcl : creatinine clearance.

## 11. Management of Candidiasis in the ICU

### 11.1. Documented Invasive Candidiasis

The time of treatment initiation is a key factor for the favorable outcome of invasive candidiasis. Several studies have shown that delay of initiation of appropriate antimicrobial therapy over 24 or 48 h has a negative impact on mortality [146,147,148]. Therefore, if candidaemia is suspected, blood cultures should be taken even in the absence of fever. In the critically ill unstable patient delays in antifungal administration predict death [149]. Consequently treatment has to be started immediately after blood cultures grow yeasts without waiting for the results of identification of *Candida* species and susceptibility tests [150]. Important considerations in the empirical choice of the right antifungal include the following: knowledge of local resistance patterns; co-morbidities of patient; presence of risk factors favoring the presence of non - *albicans* species; prior treatment with fluconazole; site of infection; spectrum of activity; known adverse effects; pharmacodynamics /pharmacokinetics; cost of treatment; but the most important factor is the presence of haemodynamic instability [114,150]. According to the current IDSA guidelines the physician can choose between fluconazole, echinocandins, amphotericin B or its lipid formulations and voriconazole (A–I) [114]. Nevertheless, the first two are considered as a preferred choice over the others. In case of (a) haemodynamic instability, (b) previous use of fluconazole or (c) isolation of *C. glabrata*, echinocandins are preferred to fluconazole. Amphotericin B is characterized as an alternative choice in case of intolerance to the other two, refractory infection, resistant organism or suspicion of infection due to pathogens other than *Candida* (e.g. cryptococcus). This is a basic difference from the guidelines of 2004 where AmB was a first choice. A step-down from echinocandin to fluconazole is suggested provided there is a clinical improvement and sterilisation of blood cultures. The recent guidelines of the European Society of Clinical Microbiology and Infectious Diseases (ESCMID 2011) [151] endorse the use of echinocandins (grade A) before LipAmB (grade B) and fluconazole (grade C). Echinocandins in patients with severe *Candida* sepsis are suggested also by Kullberg *et al.* [152] while the official statements of the American Thoracic Society suggest that in case of haemodynamic instability physicians should choose among AmB or LipAmB, echinocandins, voriconazole or high dose fluconazole [153]. Moreover Canadian guidelines published in 2010 contain specific recommendations about the critically ill [150]. According to these guidelines, fluconazole is preferred in haemodynamically stable patients with no fluconazole exposure during the last 30 days. Echinocandins are an equally accepted alternative. In case of unstable patients (with the exception of *C. parapsilosis*) an echinocandin is preferred. Finally the German speaking Mycological Society and the Paul – Ehrlich – Society for Chemotherapy suggest the use of echinocandins or liposomal amphotericin B in critically ill septic patients instead of fluconazole [154].

**Figure 1:**
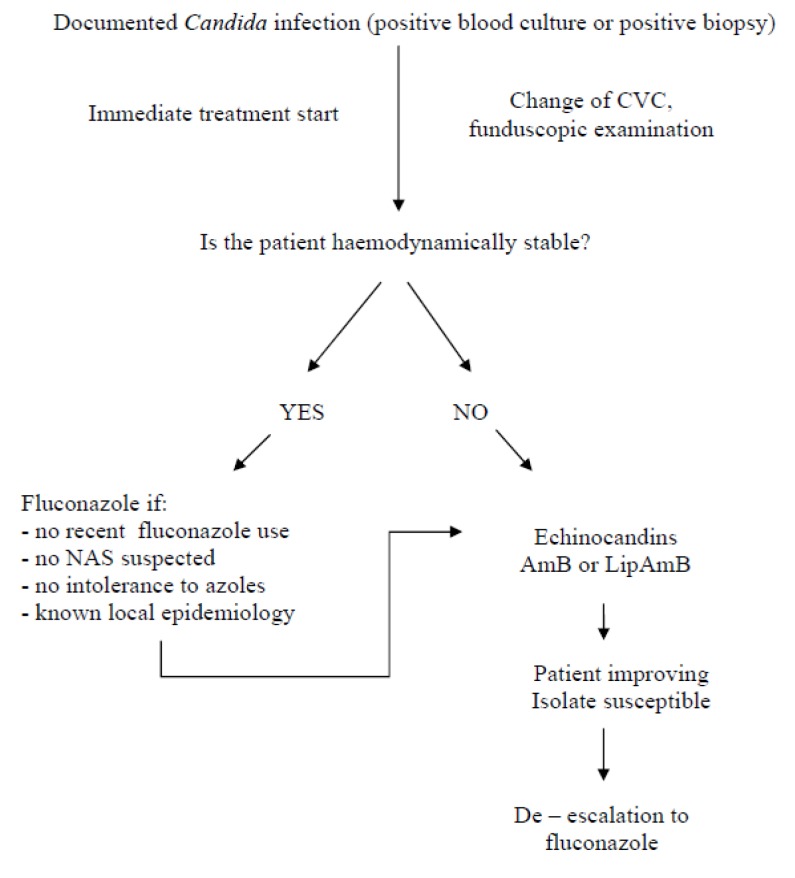
Suggested treatment algorithm for the ICU patient with invasive candidiasis (NAS: non- *albicans* species, CVC: central venous catheter, AmB: amphotericin B, LipAmB: liposomal amphotericin B).

An important parameter is that every institution should be aware of its resistance rates since in case that resistant *Candida* is common or at least not negligible, empirical treatment with azoles cannot be recommended. Local and general resistance patterns are often published [155,156] helping physicians in everyday practice particularly in the vulnerable ICU population. Comparison of the guidelines appears in Table 7 and a therapeutic algorithm is suggested in Figure 1.

**Table 7:** Suggested treatment of documented candidaemia/invasive candidiasis in non-neutropenic patients according to different guidelines.

Society	First line	Alternative I	Alternative II
**IDSA [114]**	Fluconazole	AmB or lipid formulations of AmB (intolerance to others or limited availability)	Voriconazole
	-stable patient, azole naïve		
	Echinocandins		
	-severe sepsis		
	-recent azole exposure		
**ESCMID [151]**	Echinocandins	LipAmB, voriconazole	fluconazole, lcAmB
**European Expert Opinion [152]**	Fluconazole	lipidformulations of amphotericin B	
	- stable patient		
	- susceptible isolate		
	Echinocandins		
	- severe sepsis		
	- micafungin last choice		
**Canadian clinical practice guidelines for invasive candidiasis in adults [150]**	Fluconazole	LipAmB or AmB	
	-stable patient, azole naïve		
	-unstable patient with *C.parapsilosis*		
	Echinocandins		
	-stable or unstable patient		
	-recent azole exposure		
	-avoid in *C.parapsilosis*		
**Joint recommendations of the German speaking mycological society [154]**	Fluconazole	Lip AmB -critically ill, septic patients voriconazole	
	-stable patient		
	-susceptible isolate		
	Echinocandins		
	-critically ill septic patient		

AmB = amphotericin B, LipAmB= liposomal amphotericin B, lcAmB = lipid complex amphotericin B.

Further important considerations for the practitioner include: (a) the length of therapy (b) the removal of central catheters (c) the fundoscopic examination. Serial blood cultures should be drawn after the start of treatment in order to ascertain blood sterilization. Duration of treatment is defined to 14 days after the last positive blood culture [114,150,154]. In case of persistent fungaemia, a source of local disease e.g. infective endocarditis should be sought and the presence of resistant species must be excluded. The removal of central catheters (as method of source control) is recommended by existing guidelines [114,150,154]. On the contrary some recent studies did not document any outcome improvement after the removal [157,158] while the best time for removal is not well defined, but until further data are available guidelines recommendations should be followed. Furthermore, the ability of biofilm formation in the central catheter by certain *Candida spp* supports this view. In case of catheter-related bloodstream infection, an antifungal drug with biofilm action is preferred (AmB or echinocandin). Finally, a fundoscopic examination is of pivotal importance in order to exclude disseminated endocular infection. If a form of localised candidiasis has to be treated, a longer treatment is suggested and the recommended agents are shown in Table 8.

**Table 8:** Recommended therapy for localised forms of invasive candidiasis according to 2009 IDSA guidelines.

Infection type	Suggested treatment
Pyelonephritis	fluconazole 3–6 mg/kg/d (14 days) **or** AmB 0.3–0.6 mg/kg/d for 1–7 days
Urinary fungus ball	Surgical removal recommended fluconazole 3–6 mg/kg/d **or** AmB 0.5–0.7 mg/kg/d
Candida osteomyelitis	fluconazole 6 mg/kg/d (6–12months) **or** LipAmB 3–5 mg/kg/d (weeks), then fluconazole for 6–12 months
Septic arthritis	fluconazole 6 mg/kg/d (6 weeks) **or** LipAmB 3–5 mg/kg/d (weeks) then fluconazole
CNS infection	LipAmB 3–5 mg/kg (±5FTC 25mg/kg/qid) several weeks, then fluconazole (6–12 mg/kg/d) daily **or** fluconazole 400–800 mg/d in LipAmB intolerance
Endocarditis	LipAmB 3–5 mg/kg (±5FTC 25mg/kg qid) **or** AmB 0.6–1 mg/kg/d (±5FTC 25 mg/kg) **or** an echinocandin
Suppurative thrombophlebitis	LipAmB 3–5 mg/kg/d **or** fluconazole 400–800 mg d **or** an echinocandins
Endophthalmitis	AmB 0.7–1 mg/kg plus 5FTC **or** fluconazole 6–12 mg/kg/d **or** LipAmB 3–5 mg/kg/d **or** voriconazole 6 mg/kg/12 h, then 3 mg/kg/12 h **or** echinocandins

AmB= Amphotericin B, d= daily, LipAmB= liposomal Amphotericin B, qid=4 times daily, 5FTC = flucytosine.

### 11.2. Combination Treatment

Combination of amphotericin B with flucytosine is recommended in cases of localised infection such as meningitis, osteomyelitis and intra-abdominal infections [159].

### 11.3. Other Treatment Options

Because the mortality associated with invasive candidiasis is high while the sensitivity of blood cultures is low other management options exist, including prophylactic, pre–emptive and empirical therapy.

#### 11.3.1. Prophylaxis

Prophylaxis is defined as the administration of antifungal agents to high-risk patients without signs or symptoms of infection in order to prevent the development of invasive fungal infection. The agent given systematically for that purpose is mainly fluconazole while recently the use of echinocandins has been successfully tested [160]. Although the benefits of prophylactic therapy in solid organ recipients and haematological neutropenic patients have been well established [114], in critically ill patients results are rather inconclusive because of the difficulty in the definition of high-risk patients in the heterogeneous population hospitalized in the ICU. Routine antifungal prophylaxis of all ICU patients is not recommended [150]. IDSA guidelines suggest the prophylactic use of fluconazole at a dose of 400 mg daily for high-risk ICU adult patients in hospitals with a reported high incidence of invasive candidiasis. Current literature involves several studies, meta-analyses and one Cochrane review [161,162,163,164,165,166,167,168,169,170,171]. These studies have several differences thus making the drawing of a generally applicable conclusion difficult. Most important differences include the heterogeneity in the populations examined —most include surgical ICU populations (SICU)—different doses of fluconazole used, non-similarity in the severity of underlying diseases, different incidence of invasive candidiasis and different end-points. Although some meta-analyses [168,169] concluded that fluconazole prophylaxis offered a reduction of invasive fungal infections and attributable mortality, these results cannot be easily extrapolated due to the aforementioned limitations [172]. It seems that only if the underlying risk is high (almost 10%) there is a benefit from the prophylaxis [150]. The risk is identified from the presence of risk factors described by Leon, Dupont and Ostrosky-Zeichner [56,60,61]. A reduction in fungal infections without reduction in all-cause mortality was also shown in another meta-analysis which included exclusively patients of SICUs [171]. Accordingly patients with intra-abdominal infections due to recurrent intestinal perforations or anastomotic leakage are rather a population who could benefit from this strategy while this is not the case for severe acute pancreatitis [150]. An important concern about the widespread and imprudent use of fluconazole is evidently the increase in resistance and the shift to non-*albicans* species. A more judicious approach should include the careful validation of patients at risk taking into consideration local data on rates of different *Candida* species.

#### 11.3.2. Pre-Emptive Treatment

Initiation of antifungal agents in the presence of multiple risk factors constitutes pre-emptive treatment. Prolonged ICU stay, use of broad-spectrum antimicrobials, multi-focal *Candida* colonisation, presence of gastrointestinal surgery or use of total parenteral nutrition are among the risk factors suggested for use in such cases [29,172]. The available literature about the efficacy of pre-emptive treatment is limited. In a study conducted in a surgical ICU, use of fluconazole as pre-emptive treatment in patients with a high colonisation index (according to Pittet) managed to decrease significantly the incidence of ICU-acquired invasive candidiasis without the emergence of fluconazole resistance [173]. In a recent study the monitoring of the serological marker β-d-glucan was used for identifying ICU patients at highest risk to develop an IFI and for monitoring treatment response [174]. Nevertheless existing evidence is not sufficient and more clinical investigation is required to help physicians decide which patients could benefit from pre-emptive treatment.

#### 11.3.3. Empirical Treatment

**Figure 2:**
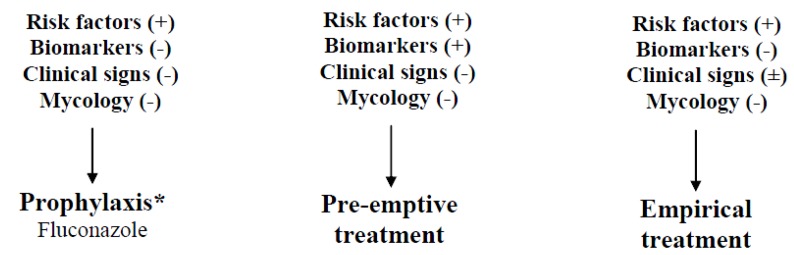
Types of treatment for suspected candidiasis in the critically ill.

If antifungal treatment is given to a patient with clinical signs of infection (e.g. persistent fever not responding to antimicrobials) and several risk factors for candidaemia without proof of invasive candidiasis, the term “empirical” may be used. Moreover this term can be applied if antifungal treatment is given to a patient with candidiasis proven by blood culture pending the results of susceptibility tests. In the first case the criteria for therapy initiation are vague in contrast to neutropenic patients and the benefits are not well established. The largest conducted study is the one by Shuster conducted in a SICU. No clear benefit of fluconazole use was proved in that study [175]. IDSA guidelines suggest that candidates for empirical antifungal therapy should be considered the critically ill patients with risk factors for invasive candidiasis and no other known cause of fever. The suggested treatment is similar to that of proven candidiasis. Main concerns about fluconazole overuse in empirical therapy include: development of resistance, therapeutic failure if (suspected) *Candida* infection is due to NAS and increasing costs [176]. Canadian guidelines suggest that critically ill patients who meet specific criteria based on clinical prediction rules may have a benefit from empirical antifungal therapy [150]. Fluconazole is suggested for stable patients and echinocandins in the presence of haemodynamic instability [150]. Figure 2 includes a suggested algorithm for the use of prophylaxis, preemptive treatment and empirical therapy.

* Prophylaxis is specific ICU population.

## 12. Treatment of Invasive Aspergillosis

Even a clinical suspicion of IPA should lead to the consideration of initiation of antifungal treatment, since the mortality of the disease remains high. AmB was the antifungal of choice for many years. However, the reported development of resistance among *Aspergillus* spp and the drug’s side effects have limited its use for the treatment of IPA [110]. Lipid-based preparations of AmB (LABA) might be considered as salvage therapy for IPA at a dose of 3–5 mg/kg/day. High doses of LABA (10 mg/kg) were proven as equally effective with lower doses (3 mg/kg/dose) while there was a trend towards more toxicity of the high dose [177].

The treatment of choice for IA is voriconazole [178]. The recommended dose is 6 mg/kg twice daily on day 1 and then 4 mg/kg/ t.i.d. The therapeutic response to voriconazole might be improved by monitoring therapeutic plasma drug levels. Another triazole, posaconazole, might be effective in IPA as salvage therapy or as prophylactic therapy in neutropenic patients. Itraconazole is an alternative option for IA refractory to AmB. Adequate absorption of the oral form should be documented by measurement of serum levels.

Other alternative drugs for the refractory cases of IPA are the echinocandins. Caspofungin has shown favorable results in patients with IA refractory to first line antifungals [179]. Micafungin and anidulafungin are not approved for IA treatment despite their activity against *Aspergillus* spp [178]*.* Combination of azoles and echinocandins might be used in refractory cases of IA [163]. The combination of voriconazole plus caspofungin compared to voriconazole led to survival advantage according to a retrospective analysis of IPA treatment [180,181]. Additionally in critically ill the combination of echinocandin with a lipid formulation of AmB might prove to be effective [181]. On the contrary the combination of voriconazole with AmB might lead to an antagonistic effect. According to IDSA guidelines combination therapy might be used in terms of salvage therapy and randomized controlled studies are indicated for further justification [178].

The duration of IPA treatment might last from several months to more than one year and should be tailored to patients’ response [178,182]. Clinical and radiological response is necessary for cessation of treatment whereas improvement of immunosuppression and sterilisation of cultures might play an important role too. Intrinsic or secondary antifungal resistance might be related to treatment failure. *A.terreus* shows primary resistance to AmB while similar susceptibility results have been demonstrated for several species of the *Fumigat*i group [179,180]. Multiple triazole resistance has increasingly been detected in *A. fumigatus* possibly due to genotypically determined resistance mechanism. Cross-resistance to triazole has also been reported. According to IDSA guidelines, itraconazole should not be used for treatment of IPA refractory to voriconazole because of the possibility of cross-resistance and the subsequent toxicity [178]. 

A reduction of the degree of immunosuppression by immunomodulatory therapy is a possible adjunctive factor to successful therapy of IPA. According to IDSA guidelines neutropenic patients with IPA might benefit from the administration of granulocyte colony-stimulating factor (G-CSF) or granulocyte-macrophage colony-stimulating factor (GM-CSF) [180]. There are also limited clinical data regarding the benefit of adding interferon-γ [183]. Granulocyte transfusion might be another adjunct to antifungals in patients with IPA but randomized control studies are lacking [168]. Finally the decrease of the dosage of systemic corticosteroids and immunosuppressive agents might contribute to a successful treatment [182]. 

Additional measures for prevention of IPA are the use of high – efficiency particulate air filtration (HEPA) and the avoidance of hospitalisation in areas with construction procedures [184]. Regarding chemoprophylaxis itraconazole or posaconazole can be used in patients with haematological malignancies and relative clinical trials are running [185].

## 13. Treatment Of Mucorales Infection

The treatment of *Mucorales* spp. requires several simultaneous approaches: aggressive (sometimes disfiguring) surgical intervention, antifungal drug therapy and management of all underlying medical conditions that might predispose the patient to the disease. On diagnosis LipAmB should be started at a dose 5–10 mg/kg once daily. The maximal dose is indicated for mucormycosis with intracerebral extension. Posaconazole, a new triazole antifungal agent, is currently considered as a second-line drug for the treatment of mucormycosis at a dose of 400 mg twice daily. Its use is recommended in combination with LipAmB or as sequential long-term therapy. Granulocyte-macrophage colony-stimulating factor (GM-CSF) and hyperbaric oxygen have been used adjunctively in neutropenic patients offering doubtful results [186].

## 14. Conclusions

Invasive fungal infections in the critical care setting often constitute a challenging diagnostic and therapeutic problem. Clinical awareness, knowledge of local epidemiology and pharmaceutical considerations are factors of paramount importance for an early diagnosis and treatment of these potentially lethal infectious diseases.
